# Programmable rotation of single cells along arbitrary axes via opto-thermo-osmotic torque

**DOI:** 10.1038/s41377-026-02424-0

**Published:** 2026-07-20

**Authors:** Siyuan Huang, Zhihan Chen, Yuebing Zheng

**Affiliations:** 1 Materials Science & Engineering Program and Texas Materials Institute, The University of Texas at Austin, Austin, TX USA; 2 Walker Department of Mechanical Engineering, The University of Texas at Austin, Austin, TX USA

**Keywords:** Optical manipulation and tweezers, Optofluidics

## Abstract

Controlled rotation of single biological cells is significant for cellular biology and engineering. Here we present a light-driven and non-contact strategy that enables arbitrary-axis rotation of both spherical and anisotropic cells with real-time switching between distinct rotation modes (major-axis and minor-axis). The platform employs a Bovine Serum Albumin (BSA)-coated gold nano-island (AuNIs) plasmonic film to generate strong interfacial thermo-osmotic flow under laser illumination, while Polyethylene Glycol (PEG)-induced depletion forces confine cells near the interface. For spherical particles, arbitrary-axis rotation is achieved using a single Gaussian beam, where spatial asymmetry in the thermo-osmotic flow determines the rotation axis. For anisotropic cells, different rotation modes are enabled by optical pattern reconfiguration. A Gaussian beam induces major-axis rotation, while a half-ring beam generates a combined optical and thermo-osmotic torque distribution that supports sustained minor-axis rotation. The rotation mode is reversibly switched solely through optical reconfiguration without mechanical intervention. This unified platform establishes geometry-independent, optically programmable rotational control, opening new opportunities for high-speed multi-angle cellular imaging and dynamic studies of cell-cell interactions.

## Abstract (web summary)

Tailored optical patterns generate reconfigurable opto-thermo-osmotic torque for programmable rotation of single cells along arbitrary axes.

## Graphical abstract

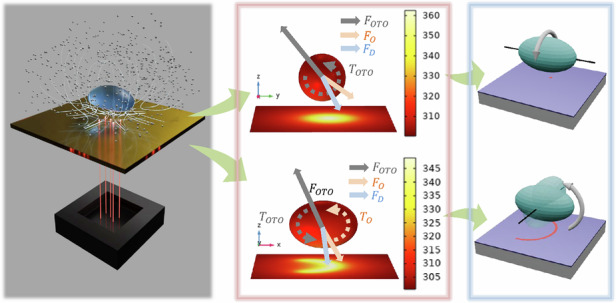

## Introduction

The precise manipulation of individual biological cells deepens understanding of cellular mechanics, morphology, and function^1–4^. Among the various manipulation techniques, controlled cell rotation stands out as a particularly powerful tool^5–9^. The ability to reorient a cell along a desired axis is a critical prerequisite for a multitude of applications. Most notably, it enables high-resolution, in-situ label-free three-dimensional (3D) imaging, where observing a cell from multiple angles is essential for reconstructing its detailed morphology without the use of potentially perturbative stains^10–16^. In addition, controlled rotation facilitates the study of intricate cell-cell interactions, allowing researchers to probe intercellular forces and communication by precisely positioning cells relative to one another^17^. The significance of these applications has driven a demand for robust and versatile cellular rotation platforms^18^.

A variety of methods for cell rotation have been explored, broadly categorized into contact-based and non-contact approaches. Contact-based methods, such as the use of micro-pipettes or mechanical probes, offer high precision but run the risk of physical damage. Non-contact methods have therefore gained prominence, leveraging different physical fields to exert forces and torques on cells, and have been increasingly explored in biomedical microrobotic systems for transporting and interacting with delicate biological samples such as cells, gametes, and embryos^19,20^. These include optoelectronic tweezers, which uses non-uniform electric fields^21,22^, magnetic rotation, which requires the internalization or attachment of magnetic beads^23,24^, acoustic tweezers, which utilize sound waves^25–27^, and chemically driven micromotors^28^. The most widely recognized non-contact technique is optical tweezing, which employs highly focused laser beams to trap and manipulate microscopic objects^29,30^. Optical tweezers can generate rotation either through the use of specially shaped beams (e.g., Laguerre-Gaussian beams) to exert direct optical torque, or by trapping and rotating indirectly via a fabricated microstructure^5,31–39^. Despite these advances, a fundamental limitation remains. Most existing approaches are optimized for rotation along a single predefined axis and often depend on particle anisotropy or specialized beam structures^29,34,35,40,41^. Arbitrary-axis rotation of spherical particles is especially challenging because isotropic geometry does not provide intrinsic directional bias. At the same time, seamless switching between rotational modes of anisotropic cells within one integrated platform has not been realized. A unified strategy that achieves programmable multi-axial rotation is therefore highly desirable. In this context, opto-thermo-osmotic mechanisms provide a distinct advantage by generating strong interfacial hydrodynamic forces, enabling geometry-independent rotation without requiring particle anisotropy or complex beam shaping.

Here we report a light-driven and non-contact platform that enables arbitrary-axis rotation of both spherical and ellipsoidal microparticles, with real-time switching between rotation modes. The platform is based on a Bovine Serum Albumin (BSA)-coated gold nano-islands (AuNIs) thin film on glass. The low zeta potential of this functionalized interface produces a large thermal slip coefficient, generating exceptionally strong thermo-osmotic flow under laser illumination^9,42–44^. Compared with conventional bulk non-isothermal convection^43,45–51^, this interfacial thermo-osmotic flow produces significantly larger hydrodynamic forces on nearby particles. Polyethylene glycol (PEG) molecules in solution introduces a depletion force that gently confines particles near the interface, where the flow is strongest^9,52^.

Using this platform, we demonstrate two rotation control schemes. For spherical particles, arbitrary-axis rotation is achieved with a single Gaussian beam, where the rotation axis is tuned by shifting the laser spot relative to the particle. Owing to particle symmetry, axis selection arises from spatial asymmetry in the thermo-osmotic flow rather than particle geometry or beam shaping, enabling geometry-independent control. For ellipsoidal cells, rotation modes are programmed by reconfiguring the optical pattern. Due to their lying configuration on the substrate, Gaussian beam produces stable rotation only along the major axis. When the beam is reshaped into a half-ring pattern, optical torque first reorients the cell, after which thermo-osmotic flow sustains rotation along the minor axis. Switching between Gaussian and half-ring patterns allows reversible transitions between rotation modes without mechanical adjustment. Numerical simulations and theoretical analysis show that spatial control of interfacial flow and optical forces enables deterministic tuning of the torque landscape.

By combining arbitrary-axis rotation of spherical particles with mode-switchable rotation of anisotropic cells, this platform provides a general approach for programmable rotational manipulation. Optical control alone determines the rotation behavior, while the system remains simple. This capability may support multi-angle label-free 3D imaging, controlled cell-cell interaction studies, and single-cell mechanical analysis^10,17^. Our results demonstrate interfacial optothermal hydrodynamics as an effective method for rotational micromanipulation.

## Results

**Fig. 1:**
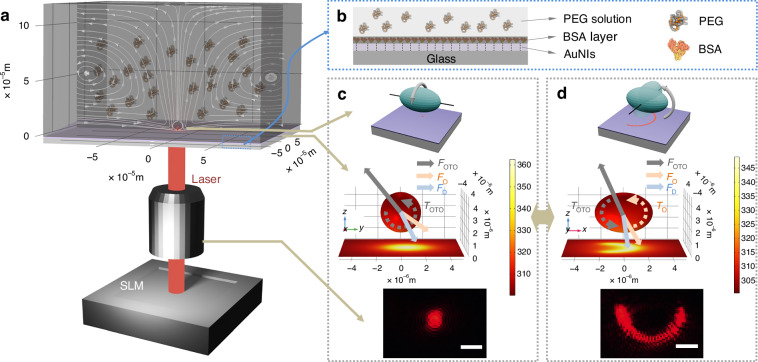
Working mechanism of cell rotation along arbitrary axes. **a** Schematic illustration of the integrated optical platform. A spatial light modulator (SLM) generates programmable laser patterns on the functionalized plasmonic substrate. The white streamlines and arrows represent the thermo-osmotic flow induced within the chamber. A detailed view of the local flow distribution is provided in Fig. 4. **b** Enlarged view of the substrate. The gold nano-islands (AuNIs) layer produces a spatially non-uniform temperature distribution under different laser illumination patterns. The AuNIs are coated with Bovine Serum Albumin (BSA), which lowers the zeta potential of the interface, thereby enhancing the thermal slip coefficient and generating strong thermo-osmotic flow. Polyethylene glycol (PEG) molecules dispersed in the aqueous solution create depletion forces under the localized temperature gradient, effectively pressing the cell toward the substrate and stabilizing its position. **c** Major-axis rotation mode. Top: Schematic of cell rotation along the major axis, the red dot indicates the laser spot position. Middle: Laser irradiation on the AuNIs layer beneath the cell generates a temperature gradient. Three dominant forces are involved: the opto-thermo-osmotic force (${F}_{{OTO}}$), the optical force (${F}_{O}$), and the depletion force (${F}_{D}$). The resulting opto-thermo-osmotic torque (${T}_{{OTO}}$) drives rotation along the major axis. Bottom: Experimental image of a single-spot Gaussian laser pattern. Scale bar, $1.7\,{\rm{\mu }}{\rm{m}}$. **d** Minor-axis rotation mode. Top: Schematic of cell rotation along the minor axis, the red curve indicates the laser pattern. Middle: Under half-ring laser illumination, the temperature distribution induces coupled opto-thermo-osmotic and optical torques (${T}_{{OTO}}$ and ${T}_{O}$), whose synergistic interaction drives rotation along the minor axis. Bottom: Experimental image of the half-ring laser pattern. Scale bar, $1.7\,{\rm{\mu }}{\rm{m}}$

The working principle of the platform is illustrated in Fig. 1a. The device consists of a sample chamber constructed on a thin glass substrate functionalized with a AuNIs layer. A 5.5 nm continuous gold film is deposited and thermally annealed to form the AuNIs structure, which serves as a plasmonic heating layer that spatially localizes laser-induced temperature fields. The AuNIs surface is subsequently coated with BSA (Fig. 1b), reducing the interfacial zeta potential to approximately -60 mV and thereby enhancing the thermal slip coefficient at the solid-liquid interface^9^. This functionalization is essential for generating strong interfacial thermo-osmotic flow under optical illumination. A 120 μm-high chamber is assembled on the functionalized substrate and filled with deionized water containing 5% PEG, in which biological cells or synthetic particles are dispersed. PEG molecules introduce a depletion force that gently confines particles near the interface without adhesion. As confirmed by persistent Brownian motion in the absence of laser illumination, particles remain suspended but stabilized within the region of strongest interfacial flow. A spatial light modulator combined with a 4 f optical system enables programmable laser pattern generation, allowing dynamic control of the temperature distribution, as shown in Figure S1.

As Fig. 1c, d shown, three dominant forces govern particle dynamics in this system: optical force, opto-thermo-osmotic force, and depletion force. The optical force provides lateral confinement, while the depletion force stabilizes the near-surface position^44,52^. Although thermo-osmotic flow produces an upward hydrodynamic component, it does not overcome the confinement, ensuring that particles remain within the region of strongest interfacial flow. This stable configuration is consistently observed for diverse biological cells as well as synthetic particles of different sizes. Upon laser illumination, the AuNIs layer produces a spatially non-uniform temperature field that drives strong interfacial thermo-osmotic flow. As Fig. 1c shown, when the laser beam is Gaussian, the resulting asymmetric flow field generates a deterministic opto-thermo-osmotic torque on particles positioned off-center relative to the heating spot. This mechanism forms the fundamental basis of rotation in our platform and applies to both isotropic and anisotropic geometries.

For spherical particles, which possess no intrinsic geometric anisotropy, the rotation axis is entirely determined by the spatial asymmetry of the thermo-osmotic flow field. By simply adjusting the lateral position of a single Gaussian beam, the direction and magnitude of the hydrodynamic torque can be continuously tuned, enabling arbitrary-axis rotation. For ellipsoidal yeast cells, the same Gaussian-beam-induced thermo-osmotic torque drives stable rotation along the major axis, as shown in Fig. 1c. In this case, the geometric anisotropy of the cell aligns the rotation with its principal axis under the asymmetric flow field. Beyond this single-mode operation, additional rotational control can be engineered through optical pattern reconfiguration. When the Gaussian beam is reshaped into a half-ring pattern, a crescent-shaped temperature distribution is generated on the substrate. The resulting torque landscape arises from the cooperative action of optical torque and thermo-osmotic torque, enabling sustained rotation along the minor axis, as illustrated in Fig. 1d. Switching between Gaussian and half-ring illumination allows real-time transitions between major- and minor-axis rotation without mechanical intervention.

For the representative yeast cell experiments, the laser power is 1.048 mW, and the absorption coefficient of the AuNIs at 660 nm is approximately 0.2. Numerical simulations of the corresponding temperature distributions are presented in Fig. 1c, d. The maximum substrate temperature reaches 362 K for the single-spot pattern and 348 K for the half-ring pattern. Importantly, owing to strong heat localization within the plasmonic layer, the maximum temperature at the surface of the suspended cell remains below 323 K (Figure S2), maintaining cell structural integrity and suggesting limited thermal perturbation during rotation. For particles and cells of different sizes and geometries, the laser power is adjusted accordingly to maintain stable rotation while preserving comparable thermal conditions.

Together, we established a general opto-thermo-osmotic framework in which spatially programmable interfacial flow generates controllable rotational torque. This mechanism enables arbitrary-axis rotation for spherical particles and mode-switchable multi-axial rotation for anisotropic cells, as systematically demonstrated in the following sections.

Here we demonstrate arbitrary-axis rotation of spherical PDMS particles, in which embedded air bubbles are used as visual markers to track particle orientation. Systematic theoretical analysis and numerical modeling are performed to elucidate the underlying rotation mechanism. Three forces govern particle dynamics in this platform: optical force, opto-thermo-osmotic force, and depletion force. The depletion force originates from the PEG distribution due to the non-uniform temperature profile^44,52^, and provides vertical confinement but does not contribute to rotational torque. Under single-spot Gaussian illumination, the optical torque is negligible, and particle rotation is dominated by the opto-thermo-osmotic torque.

**Fig. 2:**
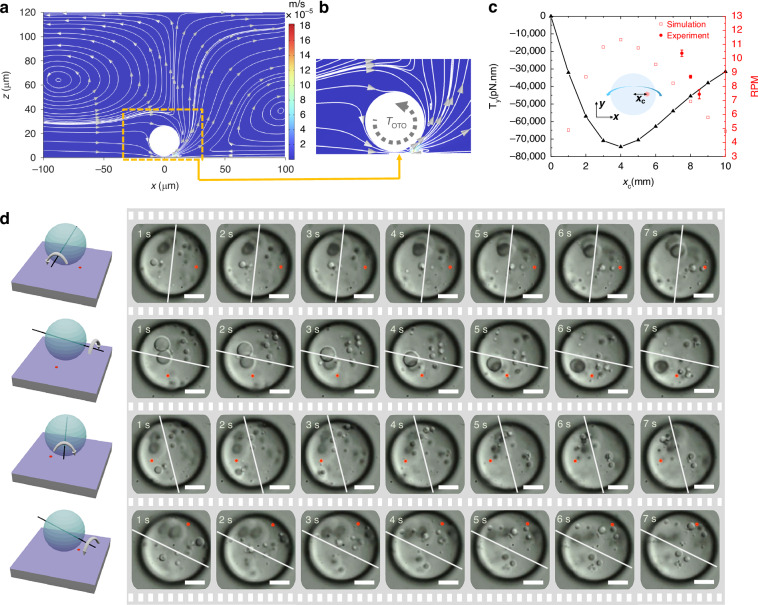
Theoretical analysis and experimental demonstration of arbitrary-axis rotation of spherical PDMS particles. **a** Simulated fluid velocity magnitude and streamlines in the chamber (x-z cross-section at y = 0 μm). The computational domain measures 200 μm (length) $\times$ 200 μm (width) $\times$ 120 μm (height). The white circle denotes the x-z cross-section of a spherical PDMS particle with a diameter of 25 μm. The color bar represents the fluid velocity (m/s). The laser spot is positioned at (x, y) = (8 μm, 0 μm)**. b** Enlarged view of the velocity distribution near the particle shown in (A). The asymmetric flow profile along the x-axis and the high-magnitude thermo-osmotic flow (on the order of tens of μm/s) give rise to a net hydrodynamic torque, which underlies the controllable rotation**. c** Calculated torque (left axis) and rotation speed (right axis) as a function of the beam-particle offset. The rotation speed includes both calculated values (from theory with wall correction) and experimental measurements. **d** Schematics and time-lapse optical images demonstrating arbitrary-axis rotation of a PDMS particle. The time interval between consecutive frames is 1 s. The red dots indicate the laser spot positions, and the white lines denote the corresponding rotation axes. Scale bar: 6.75 μm

A 3D multiphysics simulation was conducted to analyze the flow dynamics around the particle. The dimension of the computation domain was $200{\rm{\mu }}{\rm{m}}\times 200\,{\rm{\mu }}{\rm{m}}\times 120\,{\rm{\mu }}{\rm{m}}$. Figure 2a illustrates the fluid velocity intensity map and streamlines of the x-z cross-section at ${\rm{y}}=0\,{\rm{\mu }}{\rm{m}}$ in the chamber. A spherical PDMS particle with a diameter of $25\,{\rm{\mu }}{\rm{m}}$ was positioned at $({\rm{x}},{\rm{y}},{\rm{z}})\,=\,(0\,{\rm{\mu }}{\rm{m}},\,0\,{\rm{\mu }}{\rm{m}},\,13.5\,{\rm{\mu }}{\rm{m}})$. Due to the low zeta potential ($\sim -60{\rm{mV}}$) of the BSA-coated AuNIs layer^9,44^, the thermal slip coefficient of this boundary is expected to be relatively large, exceeding 0.001^43,53^. To provide a conservative estimate of the minimum torque generated by the system, we set the thermal slip coefficient to 0.001. The resulting flow velocity ${\boldsymbol{u}}$ at the BSA-coated AuNIs boundary is defined by the thermal slip boundary condition^42,43,53^:

1 $${\boldsymbol{u}}=\frac{{\sigma }_{T}\mu }{\rho T}{\nabla }_{{\rm{t}}}T$$

where ${\sigma }_{T}$ is the thermal slip coefficient, $T$ is the temperature distribution. $\mu$ and $\rho$ are dynamic viscosity and density respectively. This equation shows that the flow velocity is proportional to the temperature gradient ${\nabla }_{{\rm{t}}}T$ at the boundary. A boundary heat source was added at the BSA-coated AuNIs layer (bottom interface) to model the localized heating:

2 $${HF}\left({\boldsymbol{r}}\right)=\frac{{P}_{{HF}}}{2\pi {w}_{0}^{2}}{e}^{\frac{-{|{\boldsymbol{r}}-{{\boldsymbol{r}}}_{{\bf{0}}}|}^{2}}{2{w}_{0}^{2}}}$$

Here, ${HF}\left({\boldsymbol{r}}\right)$ is the heat flux injected through the bottom surface, ${P}_{{HF}}$ is the total power absorbed by the AuNIs layer, ${\boldsymbol{r}}$ is the position vector, ${{\boldsymbol{r}}}_{{\boldsymbol{0}}}$ is the laser spot center, and ${w}_{0}$ is the waist of the laser beam. Given the total laser power of $3.5{mW}$ and the AuNIs layer absorptivity of ~0.2 for the $660{nm}$ laser, ${P}_{{HF}}$ was set as $0.7{mW}$. The laser spot was positioned at $(x,{y})\,=\,(8\,\mu m,\,0\,\mu m)$. Further simulation parameters are provided in Table S1. As depicted in Fig. 2a, the laser positioning results in an asymmetric thermo-osmotic convection within the chamber. As shown in the enlarged view in Fig. 2b, due to the thermo-osmotic boundary condition, the peak flow rate is observed near the heat flux injection position where the temperature gradient is maximum, reaching speeds higher than $180\,{\rm{\mu }}{\rm{m}}/{\rm{s}}$. In contrast, when the boundary is modeled without the thermo-osmotic boundary condition (pure non-isothermal flow convection), the flow is on the order of ${\rm{nm}}/{\rm{s}}$ ^46^. Thus, the flow velocity is at least three orders of magnitude higher due to the thermo-osmotic effect originated from the BSA-coated AuNIs, which is consistent with our experimental observation of the rotation.

This strong thermo-osmotic flow generates a torque sufficient to drive the rotation. The total opto-thermo-osmotic torque (${{\boldsymbol{T}}}_{{\boldsymbol{OTO}}}$) on the particle is calculated by integrating the stress tensor ${\bar{\bar{{\boldsymbol{T}}}}}_{{\boldsymbol{stress}}}$ over the particle’s surface:

3 $${{\boldsymbol{T}}}_{{\boldsymbol{OTO}}}={\int }_{S}\left({\boldsymbol{r}}-{{\boldsymbol{r}}}_{{\bf{0}}}\right)\times ({\bar{\bar{{\boldsymbol{T}}}}}_{{\boldsymbol{stress}}}\cdot {\boldsymbol{n}}){dS}$$

where ${\boldsymbol{r}}$ and ${{\boldsymbol{r}}}_{{\boldsymbol{0}}}$ are the position vectors on the surface and at the center of the particle, respectively, and ${\boldsymbol{n}}$ is the outward unit normal vector on the surface. Due to the symmetry, only the ${{\rm{T}}}_{{\rm{y}}}$ component (corresponding to rotation along the y axis) is non-zero. Figure 2c plots the calculated torque ${T}_{y}$ (left axis) and the corresponding rotation speed (RPM, right axis) as a function of the lateral displacement of the laser spot from the particle center. The theoretical analysis shows that a substantial torque (on the order of ${10}^{4}{\rm{pN}}\cdot {\rm{nm}}$) can be generated, which is sufficient to drive stable rotation of the PDMS particle. The experimentally measured rotation speeds are obtained at beam-particle offsets in the range of 7.5-8.5 μm, consistent with the conditions used in Fig. 2d (Video S1). The measured rotation speeds are consistently slightly higher than the theoretical predictions, likely due to a conservative estimate of the thermo-osmotic coefficient for the BSA-coated AuNIs layer. Notably, a non-zero torque exists over a broad range of offsets, enabling stable rotation without requiring a single precise beam-particle position.

Figure 2d presents representative time-lapse optical images demonstrating arbitrary-axis rotation of a spherical PDMS particle with a diameter of 25 μm under single-Gaussian-beam illumination. By repositioning the laser spot relative to the particle center, four distinct rotation axes are selected, as indicated by the red dots (laser spot) and corresponding white lines (rotation axis). For each laser position, continuous rotation is observed, confirming stable and deterministic axis control (Video S1). Because the particle is geometrically isotropic, the rotation axis is not defined by particle shape but instead emerges solely from the spatial asymmetry of the thermo-osmotic flow field generated at the BSA-coated AuNIs interface. This demonstrates geometry-independent rotational control enabled by programmable interfacial hydrodynamics. To further examine the operational window and power dependence, additional measurements were performed using a PDMS particle with a 17 μm diameter under different laser powers (0.2 mW and 0.4 mW). As shown in Figure S3, the rotation speed increases with laser power across the entire offset range, while stable rotation is maintained over a broad range of beam-particle displacements. These experiments establish that arbitrary-axis rotation of spherical particles can be achieved using a single Gaussian beam by simply tuning the beam position, providing a fundamental basis for extending the same mechanism to anisotropic biological cells.

Having established arbitrary-axis rotation for spherical particles using a single Gaussian beam, we next extend the platform to anisotropic biological cells, where additional rotational modes become accessible through optical pattern engineering. Yeast cells were chosen as representative ellipsoidal samples to clearly demonstrate mode-switchable rotation.

**Fig. 3:**
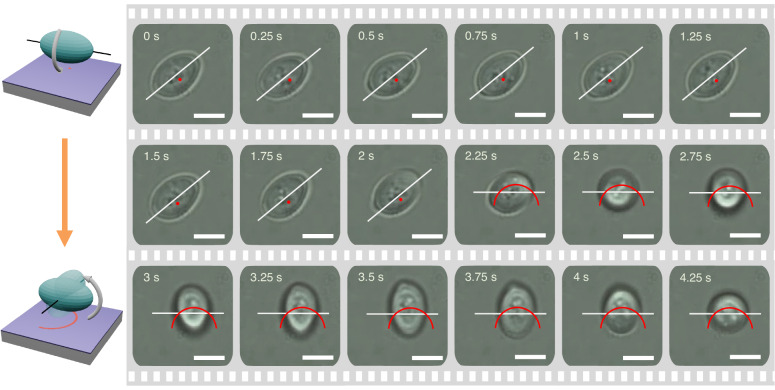
Real-time switching of rotation modes (major- and minor-axis rotation) of a yeast cell. Schematics and time-lapse optical images capturing the dynamic transition between rotation modes, with a time interval of 0.25 s between consecutive frames. Frames 1–9 show stable rotation along the major axis under a single-spot (Gaussian) laser pattern. At t = 2 s, the laser pattern is switched to a half-ring configuration. Frames 10–11 capture the transient transition between the two rotation modes. Frames 11–18 display sustained rotation of the cell along its minor axis under half-ring illumination. The red dots and curves indicate the laser patterns, and the white lines denote the corresponding rotation axes. Scale bar: 3 μm

As shown in Fig. 3, under single-spot Gaussian illumination positioned near the minor axis, the yeast cell undergoes stable rotation along its major axis (Frames 1–9). At t = 2 s, the laser pattern is dynamically reconfigured into a half-ring profile, triggering a rapid transition between rotation modes. Within 0.5 s, the cell reorients and enters sustained minor-axis rotation (Frames 11–18). This real-time switching is fully reversible, as further demonstrated in Video S2, where repeated transitions between major- and minor-axis rotation are achieved solely through optical reconfiguration, without mechanical adjustment or sample manipulation. The rotation along the major axis is observed during 1^st^ to 20^th^ s and 42^nd^ to 67^th^ s, while the rotation mode is switched to the minor axis during 20^th^ to 42^nd^ s and 67^th^ to 80^th^ s.

To verify the generality of this approach, we additionally demonstrate mode-switchable rotation of algae cells (Video S3). Although algae exhibit weaker geometric anisotropy than yeast, distinct rotation axes are readily identified by tracking the ellipsoidal nucleus. Consistent with the yeast experiments, both major- and minor-axis rotations are achieved by switching between Gaussian and half-ring illumination. In Video S3, the laser pattern was initially set as a half ring and was switched to a single spot at 24^th^ s. Owing to the smaller size of algae cells, the half-ring radius is reduced to 1.4 μm and the laser power is adjusted to 0.69 mW to maintain comparable thermal conditions.

Together, these results demonstrate real-time and reversible multi-axis rotation of anisotropic biological cells. Combined with the geometry-independent control established for spherical particles, this platform provides a unified and programmable strategy for arbitrary-axis rotation across diverse cellular geometries.

Building on the opto-thermo-osmotic torque established for spherical particles in Fig. 2, we quantitatively analyze the rotational dynamics of ellipsoidal cells to elucidate both major- and minor-axis rotation mechanisms. 3D multiphysics simulations were performed. Further simulation parameters are provided in Table S1. Consistent with the spherical particle case, optical torque is negligible under single-Gaussian-beam illumination, and rotation is primarily governed by opto-thermo-osmotic torque.

**Fig. 4:**
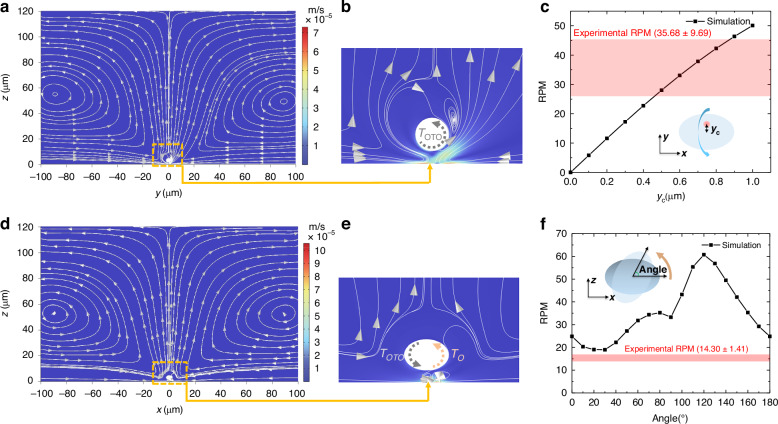
Theoretical analysis of major- and minor-axis rotation of an ellipsoidal cell model. **a** Simulated fluid velocity magnitude and streamlines in the chamber (y-z cross-section at x = 0 μm). The white circle denotes the y-z cross-section of the ellipsoid (major axis: 2.45 μm; minor axis: 1.7 μm). The color bar indicates fluid velocity (m/s). The laser spot is positioned at (x, y) = (0 μm, 0.5 μm). **b** Enlarged view of the near-field velocity distribution from **a**, showing asymmetric thermo-osmotic flow along the y-axis that drives rotation along the major axis (x-axis). **c** Theoretical rotation speed (RPM) as a function of beam-particle offset ${{\rm{y}}}_{{\rm{c}}}$ along the y direction (x = 0 μm), obtained from the simulated torque using near-wall hydrodynamic correction. The red shaded region indicates the experimentally measured RPM (mean ± standard deviation, 35.68 ± 9.69 RPM). **d** Fluid velocity map and streamlines (x-z cross-section at y = 0 μm) under half-ring illumination centered at (-1.6 μm, 0 μm) with a radius of 2.3 μm. The white circle represents the ellipsoid cross-section. **e** Enlarged near-field velocity distribution corresponding to **d**. **f** Theoretical rotation speed (RPM) as a function of the rotation angle for minor-axis rotation. The red shaded region represents the experimentally measured RPM (mean ± standard deviation, 14.30 ± 1.41 RPM)

As shown in Fig. 4a, b, when a Gaussian beam is displaced along the minor axis by $0.5\,{\rm{\mu }}{\rm{m}}$ relative to the cell center, asymmetric interfacial thermo-osmotic flow is generated, with peak velocities exceeding $70\,{\rm{\mu }}{\rm{m}}/{\rm{s}}$ near the substrate. Figure S4 shows the fluid velocity map of the x-z cross-section at different y positions, demonstrating a strong flow rate exceeding $30\,{\rm{\mu }}{\rm{m}}/{\rm{s}}$ directly underneath the ellipsoid. Integration of the hydrodynamic stress over the ellipsoid surface yields an opto-thermo-osmotic torque sufficient to drive stable major-axis rotation under experimental conditions. The corresponding rotation speeds, obtained from theoretical prediction with near-wall correction, are consistent with experimental measurements (Fig. 4c). This agreement confirms that major-axis rotation of ellipsoidal cells originates from the same thermo-osmotic mechanism governing spherical particles. The detailed torque analysis is provided in Figure S5.

The mechanism underlying minor-axis rotation is fundamentally different from that of major-axis rotation. In our geometry, the opto-thermo-osmotic torque cannot sustain minor-axis rotation because the ellipsoidal cell preferentially relaxes to a lying configuration with its major axis parallel to the substrate. To overcome this geometric restoring tendency, we engineer a cooperative torque landscape by switching the illumination to a half-ring pattern, where optical torque assists cell reorientation and opto-thermo-osmotic torque drives the remaining rotation.

**Fig. 5:**
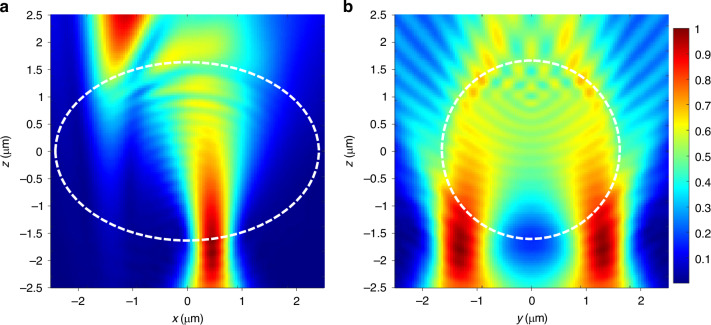
Normalized electric field distribution around the ellipsoid when the laser pattern is set as a half-ring (Angle=0° case). **a** Shows the x-z cross-section taken at y = 0 μm. **b** Shows the y-z cross-section taken at x = 0 μm

In the opto-thermo-osmotic simulation for the minor-axis rotation mode, the half-ring illumination is modeled as a superposition of N Gaussian beams, with the boundary heat flux defined as:

4 $${HF}\left({\boldsymbol{r}}\right)=\mathop{\Sigma }\limits_{i=1}^{N}\frac{{P}_{{HF}}}{2\pi N{w}_{i}^{2}}{e}^{-\frac{{|{\boldsymbol{r}}-{{\boldsymbol{r}}}_{{\boldsymbol{i}}}|}^{2}}{2{w}_{i}^{2}}}$$

where $N=30$ matches the experimental pattern, ${{\boldsymbol{r}}}_{{\boldsymbol{i}}}$ denotes the center of the ${i}^{{th}}$ beam, and the total absorbed power ${P}_{{HF}}$ is set to $0.2096\,{\rm{mW}}$. The resulting flow field (Fig. 4d, e) exhibits strong asymmetry near the substrate with peak velocities exceeding $100\,\mu m/s$. Figure S6 shows the fluid velocity map of the y-z cross-section at various x positions, with flow rates consistently above $30\,\mu m/s$ around the ellipsoid. This structured opto-thermo-osmotic flow produces an angle-dependent thermo-osmotic torque along the minor axis. As detailed in Figure S7, torques at various angles are calculated by incorporating the tilted angle of the ellipsoid into the geometry configuration and recalculating the flow distribution. We then compute the optical torque under the same half-ring illumination by first obtaining the electromagnetic fields using FDTD (Fig. 5), and evaluating the Maxwell stress tensor^54^:

5 $$\begin{array}{l}{\bar{\bar{{\boldsymbol{T}}}}}_{{\boldsymbol{M}}}=\\\frac{1}{2}\mathrm{Re}\left[{\epsilon }_{m}{\boldsymbol{E}}\otimes {{\boldsymbol{E}}}^{\star }+{\mu }_{m}{\boldsymbol{H}}\otimes {{\boldsymbol{H}}}^{\star }-\frac{1}{2}\left({\epsilon }_{m}{|{\boldsymbol{E}}|}^{2}+{\mu }_{m}{|{\boldsymbol{H}}|}^{2}\right)\bar{\bar{I}}\right]\end{array}$$

where ${\epsilon }_{m}$ and ${\mu }_{m}$ are the relative permittivity and permeability, respectively. The resulting optical torque is then calculated by integrating the stress tensor over the entire ellipsoid:

6 $${{\boldsymbol{T}}}_{{\boldsymbol{O}}}=\int \nabla \cdot \left({\bar{\bar{{\boldsymbol{T}}}}}_{{\boldsymbol{M}}}\times {\boldsymbol{r}}\right){dV}$$

The calculated optical torque and opto-thermo-osmotic torque exhibit opposite angular dependencies, as shown in Figure S5B (blue and red curves). Their sum yields a total torque that remains negative over the full rotation cycle (black curve), which explains the sustained anticlockwise minor-axis rotation observed experimentally. The theoretical rotation speed, derived from the combined torque with near-wall hydrodynamic correction, varies with the rotation angle and predicts continuous rotation without equilibrium points (Fig. 4f). Experimentally, the rotation speed is obtained by measuring the time required for a full rotation cycle, yielding a distribution of average RPM values. Although the theoretical prediction overestimates the rotation speed, it captures the absence of equilibrium configurations and the continuous rotational behavior observed in experiments. The quantitative discrepancy likely arises from the quasi-static nature of the model, which does not fully capture the unsteady hydrodynamic perturbations generated during minor-axis rotation. Unlike major-axis rotation, minor-axis rotation continuously changes the cell orientation and the local fluid flow configuration near the interface, leading to additional viscous dissipation and an increased rotational drag.

To further support the proposed mechanism, we performed a control experiment on a bare glass substrate under identical half-ring illumination. The yeast cell reorients from a horizontal configuration to an upright state but remains stationary thereafter, without sustained rotation (Figure S8 and Movie S4), indicating that optical torque alone is insufficient to drive continuous rotation. Notably, optical torque dominates for rotation angles below 90°, lifting the cell toward an upright configuration, whereas opto-thermo-osmotic torque dominates beyond 90°, completing the rotation cycle (Figure S5). This complementary interplay between optical lifting and opto-thermo-osmotic driving establishes a continuous rotation cycle and enables stable minor-axis rotation.

## Discussion

By harnessing the synergistic interplay between opto-thermo-osmotic torque and optical torque, we achieve deterministic arbitrary-axis rotation of both spherical particles and anisotropic biological cells, together with real-time and reversible switching between distinct rotation modes, a capability that remains challenging for existing rotational manipulation techniques. Systematic theoretical analysis and numerical modeling quantitatively reveal the mechanisms governing both major- and minor-axis rotation. Crucially, rotational control is realized solely through optical reconfiguration, without mechanical intervention or particle modification. Recent advances in 3D opto-hydrodynamic manipulation, including strategies based on optically driven colloidal assemblies, have also demonstrated versatile rotational control^55^. Our approach complements these efforts by enabling programmable rotation through direct optical reconfiguration within a single-particle framework.

Beyond the Gaussian and half-ring patterns demonstrated here, the intrinsic programmability of optical fields offers a broader opportunity for torque landscape engineering. By designing tailored illumination geometries, the cooperative action of optical and opto-thermo-osmotic torques may enable richer rotational dynamics, providing a versatile framework for future micromechanical manipulation and programmable rotational control. In addition, while the current implementation relies on a bulk optical setup, the underlying concept of optically programmable torque landscapes is compatible with future photonic integration. Emerging platforms such as metasurfaces and integrated phase modulators may enable compact and scalable implementations of dynamic beam shaping and switching^56,57^. Coupling such photonic elements with microfluidic systems could provide a pathway toward fully integrated and modular devices for programmable micromanipulation. Furthermore, the use of spatial light modulation allows extension to parallel manipulation via multi-spot or patterned illumination, providing a pathway toward high-throughput applications.

Owing to the low optical power required, this platform holds strong potential for label-free 3D imaging and dynamic studies of cell-cell interactions^10,17^. While no observable structural damage was detected during repeated rotation cycles, long-term viability and functional effects remain to be systematically investigated. Nevertheless, controlled rotation naturally complements confocal and structured illumination microscopy by enabling continuous 360° observation of intracellular structures, thereby improving the fidelity and completeness of three-dimensional reconstruction^58–61^. In addition, coupling with Raman spectroscopy, fluorescence lifetime imaging microscopy, or optoacoustic imaging may allow real-time, orientation-dependent probing of molecular composition and cellular microenvironments^62–65^, opening new opportunities for non-invasive cell characterization, mechanobiology studies, pathology assessment, and dynamic evaluation of drug-cell interactions.

## Materials and Methods

### Materials

Polyethylene glycol (PEG, Mw 20,000) and bovine serum albumin (BSA) were purchased from Sigma-Aldrich. Saccharomyces cerevisiae (active dry yeast, Fleischmann’s Yeast, AB Mauri) and Chlorella vulgaris (Carolina Biological Supply Company) were used in this study. Deionized water was produced by Barnstead Smart2Pure Water Purification System (Thermo Scientific). Polydimethylsiloxane (PDMS, Sylgard 184) was purchased from Dow Corning.

### Sample preparation

Device preparation: Au nano-island (AuNIs) substrates were fabricated starting from standard glass slides. A thin Au film (5.5 nm) was deposited onto the glass substrates by electron-beam evaporation at a base pressure of $8\times {10}^{-6}$ Torr. The as-deposited Au films were subsequently annealed in air at 550 °C for 2 h to form nano-island structures. After cooling to room temperature, the substrates were immersed in a 1 wt% bovine serum albumin (BSA, Sigma-Aldrich, A8531) aqueous solution for 24 h to achieve surface functionalization. The samples were then thoroughly rinsed with deionized (DI) water to remove loosely bound molecules and dried under a nitrogen stream before use. A schematic illustration of the fabrication process is provided in the Supporting Information (Figure S9). A 5 wt% PEG 20000 powder (Sigma-Aldrich, 8.18897) and a 1% particle or cell solution were dissolved in DI water to obtain the targeted PEG solution. A 16 μL PEG solution with cells was added on the prepared BSA-coated AuNIs substrate with a spacer (Secure-Seal), and then an 18 × 18 mm^2^ coverslip (Thermo Fisher Scientific) was placed on the top, creating a liquid film (thickness: 120 μm) between the coverslip and the substrate.

Synthesis of PDMS particles: PDMS base and curing agent were mixed at a weight ratio of 10:1 and dispersed into an aqueous solution containing 1 wt% PEG 20000. The mixture was stirred at 1500 rpm for 10 min to form an emulsion, followed by heating to 45 °C while reducing the stirring speed to 400 rpm for 1.5 h. The emulsion was then left at room temperature for 12 h to allow complete curing of the PDMS microparticles.

### FEA simulation

The Finite Element Analysis (FEA) software COMSOL Multiphysics version 6.0 was employed to simulate the temperature distribution and the resulting thermo-osmotic flow. The simulation coupled the Laminar Flow module and the Heat Transfer in Solids and Fluids module using the Nonisothermal Flow Multiphysics interface. Laser heating was modeled as a heat flux defined at the bottom boundary of the simulation domain. This structured heating source was defined using the specific laser beam profiles (single-spot or half-ring pattern) along with input parameters for laser power, beam width, and material absorptivity. A spherical or ellipsoidal particle was introduced near the substrate within the fluid domain, with detailed modeling parameters provided in Table S1. Boundary conditions were defined as follows: the bottom boundary (substrate interface) was modeled as a thermal slip boundary with a thermal slip coefficient of 0.001. The upper boundary was set as a wall with a no-slip condition, while the side boundaries were defined as an open boundary. Finally, the thermo-osmotic flow-induced torque was calculated via post-treatment of the stress tensor results using Eq. 3.

### FDTD simulation

The optical field distribution was calculated using the Finite-Difference Time-Domain (FDTD) software Lumerical. Detailed simulation parameters are provided in Table S1. To ensure accurate modeling of the open boundaries, Perfectly Matched Layer (PML) boundary conditions were applied in all directions of the simulation domain. The simulation time was sufficient to ensure that the field energy decayed below ${10}^{-5}$. The resulting electric field and magnetic field data were then exported and analyzed using a custom-developed MATLAB code to calculate the optical torque exerted on the ellipsoid.

### Optical setup

The optical configuration used in our experiment is schematically illustrated in Fig. S1. A red laser ($\lambda =660{nm}$, Laser Quantum, Opus 660) is first expanded by a factor of 5. The expanded beam is then directed toward a liquid crystal on silicon-spatial light modulator (SLM; Hamamatsu, X13138-01), which has a resolution of $1392\times 1040$ pixels. The incident laser beam was linearly polarized using a polarizer placed before the spatial light modulator (SLM), with the polarization aligned to the liquid-crystal orientation to ensure efficient phase-only modulation. No additional polarization element was used after the SLM, and the polarization state was maintained throughout the optical path. The SLM diffracts the laser beam according to holographic patterns generated by a computer. The diffracted laser pattern subsequently passes through a $4f$ lens setup (with an image reduction factor ${f}_{1}/{f}_{2}\,=\,0.75$) before entering an inverted optical microscope (Nikon, Ti2). For illumination and observation, we use an oil-immersion objective lens ($60\times$ magnification; Nikon, CFI Plan Fluor 60XS Oil). Optical images and videos of the experiment are captured using a charge-coupled device (CCD) camera (Lumenera, INFINITY 2).

### Hologram generation

The optical fields were generated using a phase-only spatial light modulator (SLM) controlled by the Red Tweezers platform (LabVIEW). The holograms were calculated using the direct superposition (gratings and lenses) algorithm, in which the complex fields corresponding to individual trap positions are summed to obtain the final phase distribution. The Gaussian beam corresponds to a single trap, while the half-ring beam was generated by defining multiple trap coordinates arranged along a half-ring geometry. The SLM patterns were updated in real time with an update interval of 0.125 s (8 Hz).

## Supplementary information

Supplementary Information

Video S1

Video S2

Video S3

Video S4

## Data Availability

All data needed to evaluate the conclusions in the paper are present in the paper and/or the Supplementary Materials.
