# Supplementary material for: Programmable rotation of single cells along arbitrary axes via opto-thermo-osmotic torque
Source: Light Sci Appl. 2026 Jul 20;15:325. doi: 10.1038/s41377-026-02424-0 (PMC13381599; doi:10.1038/s41377-026-02424-0)
Supplement: Supplementary file 1 — Supplementary Information [file 41377_2026_2424_MOESM1_ESM.pdf]

Supplementary Information for

Programmable Rotation of Single Cells Along Arbitrary Axes via Opto-thermo-osmotic Torque

Siyuan Huang[1], Zhihan Chen[1], and Yuebing Zheng[1,2*]

[1]Materials Science & Engineering Program and Texas Materials Institute, The University of Texas at Austin, Austin, TX 78712, USA.

[2]Walker Department of Mechanical Engineering, The University of Texas at Austin, Austin, TX 78712, USA.

*Yuebing Zheng

Email: zheng@austin.utexas.edu

This PDF file includes:

Supplementary Text S1

Figures S1 to S10

Table S1

Other supporting materials for this manuscript include the following:

Movies S1 to S4

Supplementary Text

S1. Determination of experimental and theoretical RPM

The experimental rotation speed of the PDMS particle was obtained by tracking the particle center (x_c, y_c) and the position of a bubble at the particle edge (x_b, y_b). The relative coordinates were first calculated as:

$$\mathit{\Delta} x(t) = x_b(t) - x_c(t), \mathit{\Delta} y(t) = y_b(t) - y_c(t)$$

Because the rotation axis lies in the x-y plane, the projected bubble trajectory follows a line path. To extract the rotational motion, the relative trajectory $(\Delta x, \Delta y)$ was first centered by subtracting its mean value and then projected onto its dominant direction using principal component analysis (PCA), yielding a one-dimensional signal $s(t)$. The analytic signal was constructed as:

$$z(t) = s(t) + i\mathcal{H}[s(t)]$$

Where $\mathcal{H}[\cdot]$ denotes the Hilbert transform. The phase $\phi(t)$ was then obtained as

$$\phi(t) = unwrap(\arg z(t))$$

And the angular velocity was calculated as:

$$\omega(t) = \frac{d\phi(t)}{dt}$$

The rotation speed was then given by:

$$RPM(t) = \frac{\omega(t)}{2\pi} \times 60$$

And the reported RPM corresponds to the time-average value.

For the ellipsoidal yeast cell, the rotation speed was determined using a frame-counting method instead of continuous phase tracking. Due to the small particle size and limited spatial resolution, the instantaneous orientation angle could not be reliably extracted. Therefore, the rotation period was obtained by counting the number of frames required for one full rotation cycle. Specifically, a rotation cycle was defined based on the recurrence of the projected cell configuration (identical contour). If N frames correspond to one full rotation, the rotation speed is given by $RPM = \frac{60}{N\Delta t}$, where Δt is the time interval between consecutive frames. Multiple rotation cycles were analyzed

to obtain a set of RPM values. The statistical distribution of the measured rotation speeds for both major- and minor-axis rotation is summarized in Figure S10.

For the theoretical prediction, the simulated torque T was converted to rotation speed using a near-wall hydrodynamic correction for a sphere rotating close to a boundary [1]:

$$RPM = \frac{60}{2\pi} \frac{10^{-21} \cdot T}{8\pi\mu a^3[0.4 \ln\left(\frac{a}{g}\right) + 0.3709]}$$

Where T is in $pN \cdot nm$, μ is the viscosity, a is the particle radius, and g is the particle-wall gap.

For the ellipsoidal cell, the rotational drag was generalized to that of a prolate spheroid:

$$\zeta = 8\pi\mu R_{eff}^3 F, R_{eff} = (ab^2)^{\frac{1}{3}}$$

Where a and b are the semi-axes and F is the Perrin rotational friction factor, which depends on the rotation axis. The near-wall correction was incorporated in the same form, yielding

$$\zeta_{wall} = 8\pi\mu R_{eff}^3 F[0.4ln\left(\frac{R_{wall}}{g}\right) + 0.3709]$$

Where R_{wall} is the characteristic radius associated with the rotation axis (taken as b for major-axis rotation and $\sqrt{ab}$ for minor-axis rotation). The rotation speed was then obtained from:

$$RPM = \frac{60}{2\pi} \cdot \frac{10^{-21}T}{\zeta_{wall}}$$

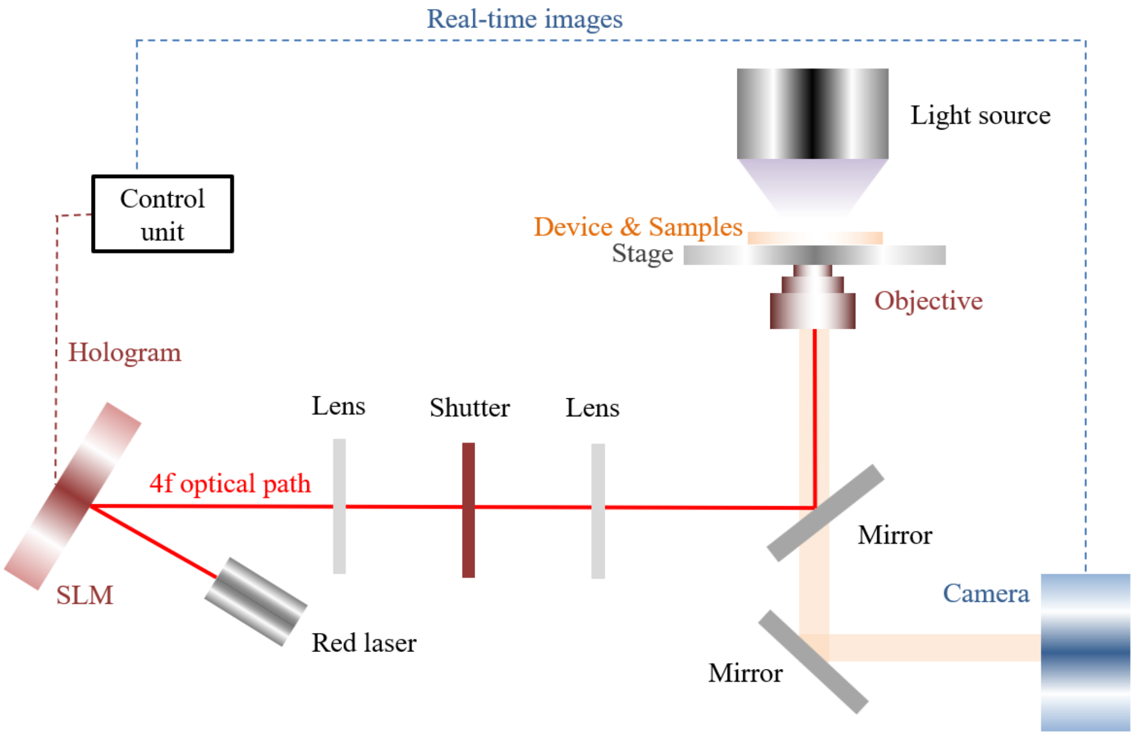

Figure S1. Schematic diagram of the experimental setup. This diagram illustrates the experimental configuration used for generating and controlling the rotation modes. A Spatial Light Modulator (SLM), integrated with a 4f optical path, is utilized for laser pattern generation. Videos of the resulting particle motion are captured by a camera. The system is controlled by a LabVIEW program, which handles the hologram writing and provides the necessary control for the real-time switching between different rotation modes.

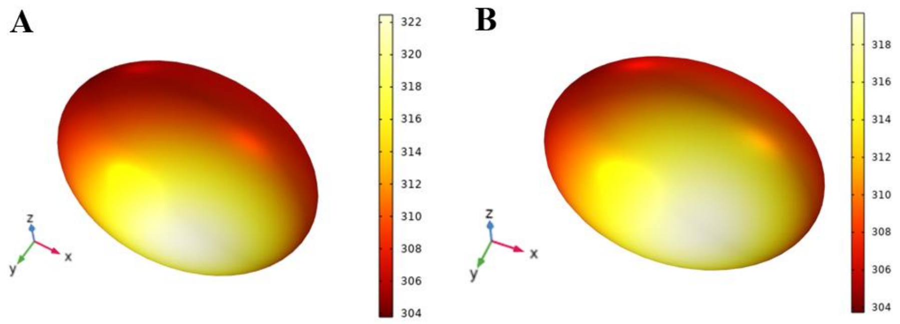

Figure S2. Theoretical temperature distribution around the ellipsoid particle when subjected to two different laser patterns. (A) Temperature distribution under the single-spot laser pattern. (B) Temperature distribution under the half-ring laser pattern.

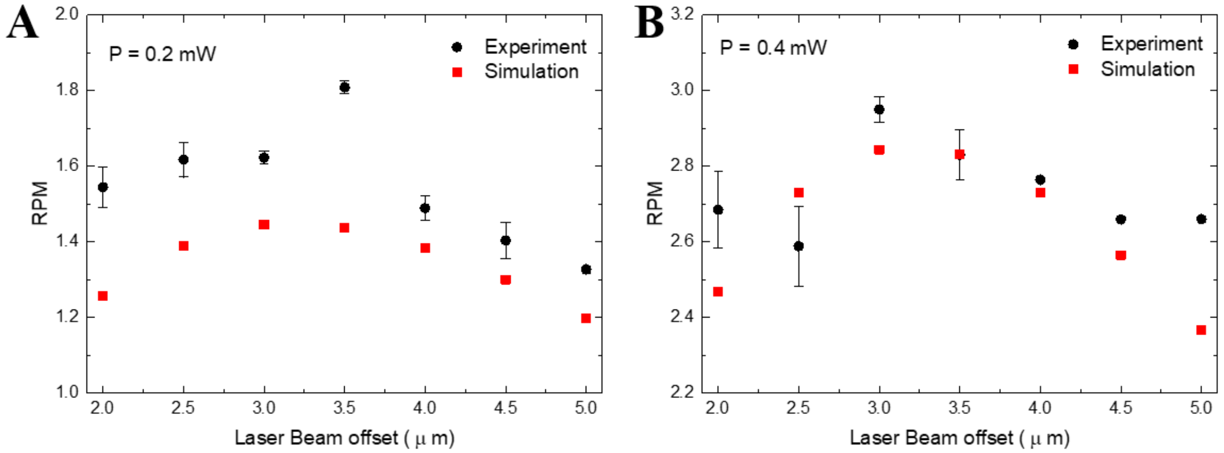

Figure S3. Rotation speed (RPM) of a 17 μm PDMS particle as a function of beam-particle offset under two laser powers (0.2 mW and 0.4 mW). Experimental results (black circles) are compared with simulations (red squares), including wall-correction. Increasing laser power leads to higher rotation speeds over the entire offset range, consistent with enhanced opto-thermo-osmotic torque.

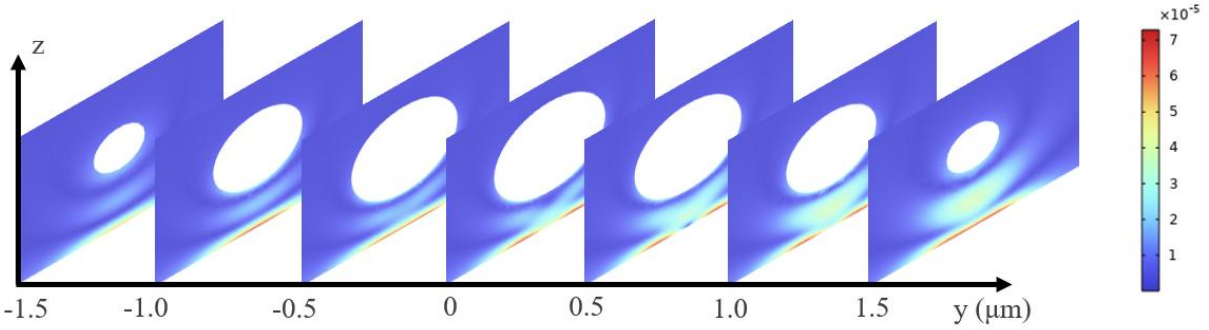

Figure S4. Fluid Velocity Map of the x-z cross-section at various y positions when the laser pattern is a single spot, illustrating the flow dynamics responsible for rotation along the major axis. The color bar indicates the fluid velocity in m/s.

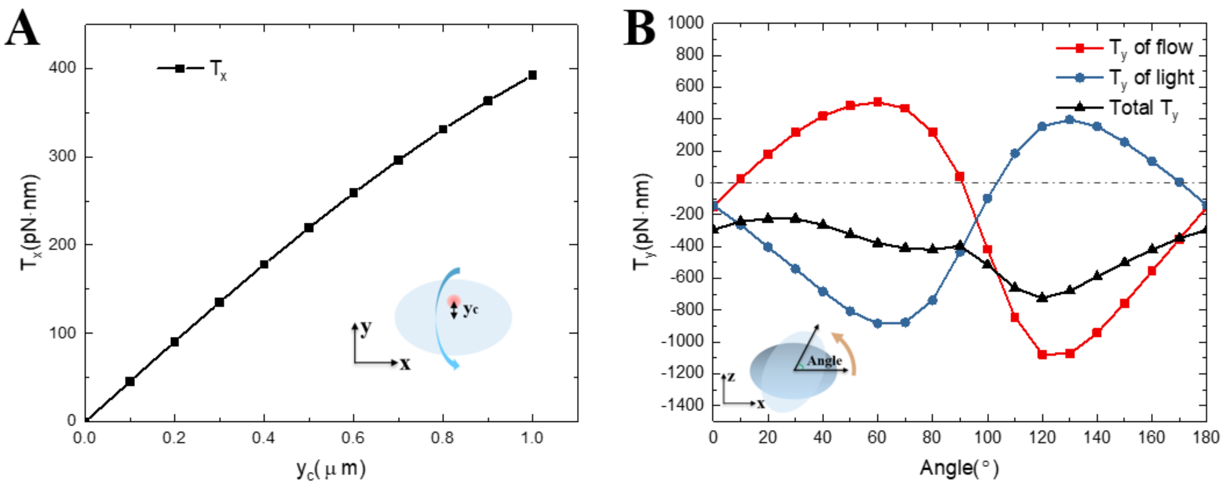

Figure S5. Calculated torque. (A) Calculated torque along the major axis (x-axis) as a function of the beam-particle offset yc along the y direction (x = 0 μm). (B) Calculated torque along the minor axis (y-axis) as a function of the ellipsoid rotation angle. The red, blue, and black curves denote the opto-thermo-osmotic torque, optical torque, and total torque, respectively.

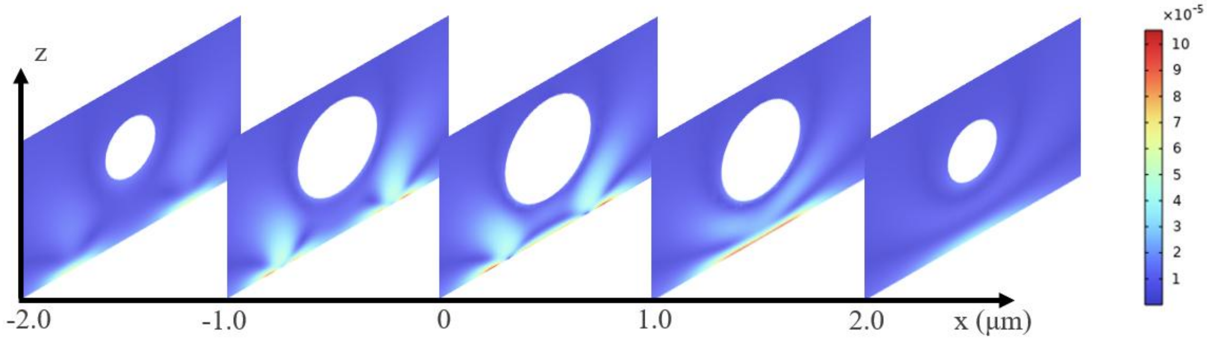

Figure S6. Fluid Velocity Map of the y-z cross-section at various x positions when the laser pattern is a half ring, illustrating the flow dynamics responsible for rotation along the minor axis. The color bar indicates the fluid velocity in m/s.

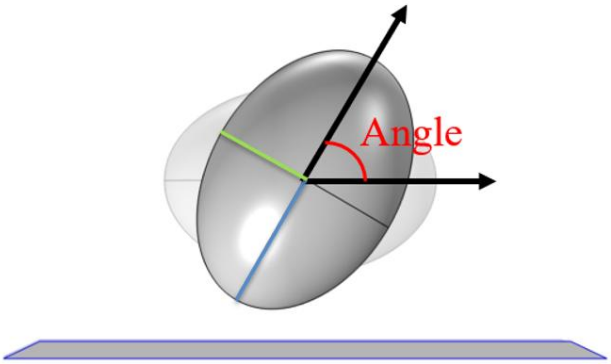

Figure S7. Schematic representation of the different rotational angles of the ellipsoid employed in the COMSOL simulation configuration for calculating the torque and fluid dynamics results shown in Figure 4 (Rotation along minor axis). Blue line and green line indicate the major axis (2.45 μm) and minor axis (1.7 μm) respectively.

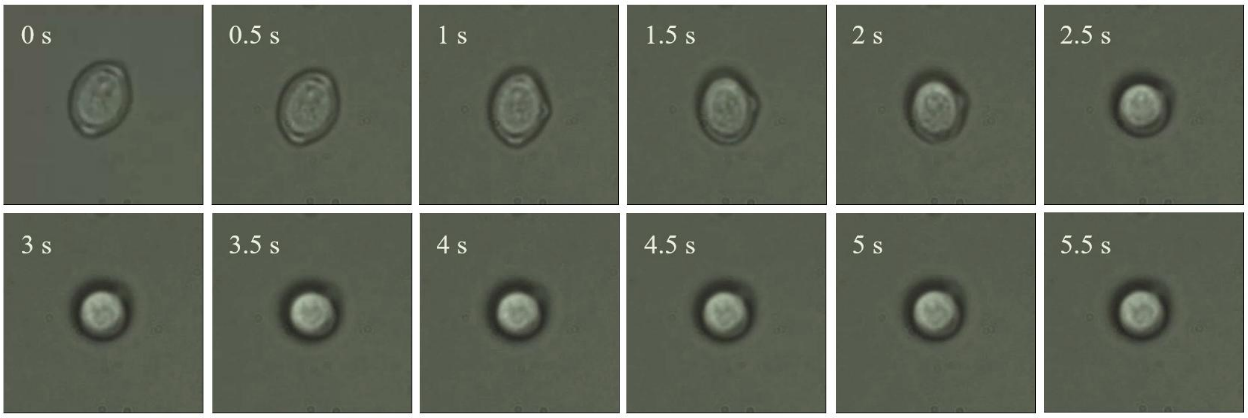

Figure S8. Reorientation of a yeast cell under half-ring illumination on a bare glass substrate. Time-lapse images showing the evolution of a yeast cell in 5% PEG solution under half-ring optical pattern. The cell reorients from a horizontal configuration to an upright state and remains stable thereafter without continuous rotation. The absence of sustained rotation in this control experiment suggests that optical torque can induce cell reorientation, whereas continuous minor-axis rotation requires the additional opto-thermo-osmotic flow generated on the BSA-coated AuNIs substrate.

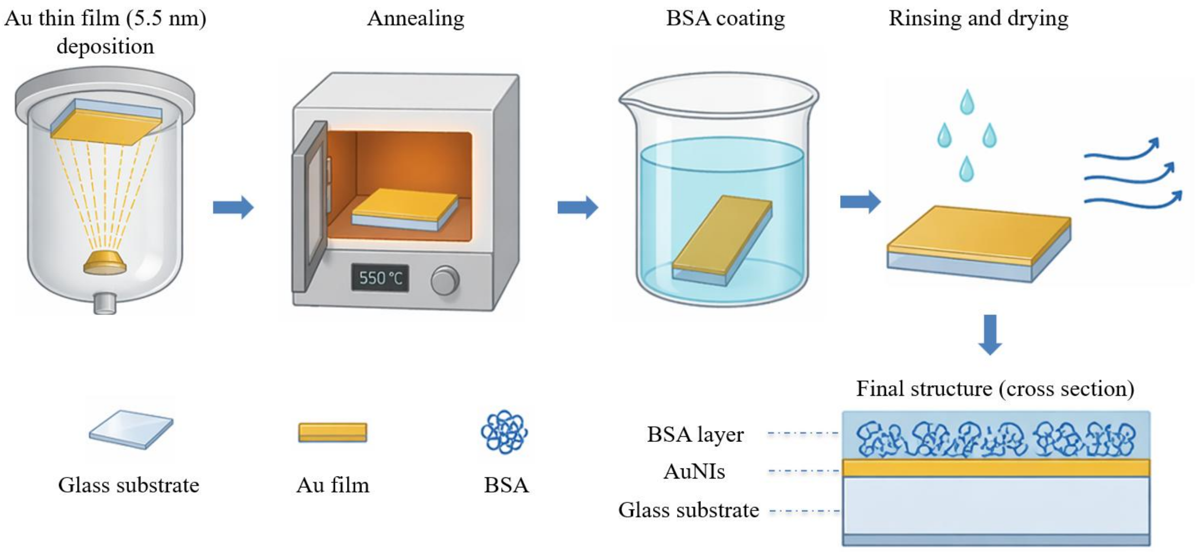

Figure S9. Schematic illustration of the fabrication process of the BSA-coated Au nano-islands (AuNIs) substrate. A 5.5 nm Au film is first deposited on a glass substrate via e-beam evaporation, followed by thermal annealing at 550 °C to form nano-islands. The substrate is then immersed in a 1% BSA solution for surface functionalization, rinsed with deionized water, and dried under nitrogen. A cross-sectional view of the final structure is shown on the right.

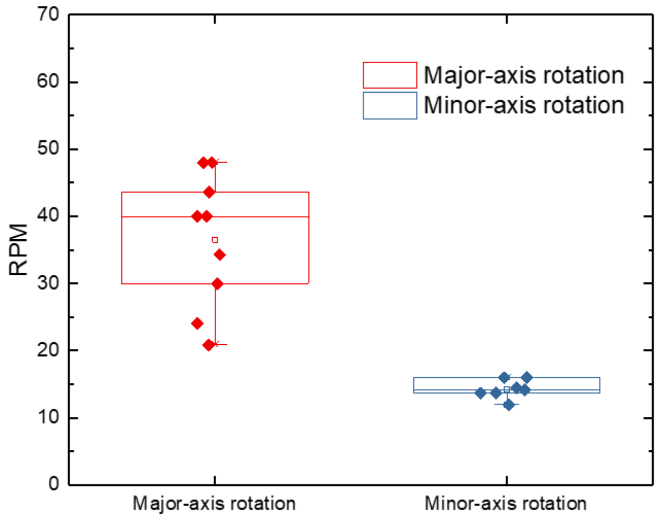

Figure S10. Statistical distribution of experimentally measured rotation speeds. Box charts of the rotation speed (RPM) for major-axis rotation (n = 9) and minor-axis rotation (n = 7). Individual measurements are overlaid as scatter points. Each data point corresponds to the rotation speed obtained from an individual rotation cycle.

Table S1. Detailed parameters for the FEA and FDTD simulation.

FEA	
Software	COMSOL
P_{HF} for PDMS particle	0.7 mW
P_{HF} for Ellipsoid	0.2096 mW
Dimension of simulation domain (Figure 2 & 4)	$200\ \mu m \cdot 200\ \mu m \cdot 120\ \mu m$
Radius of PDMS particle	25 μm
Size of the ellipsoid	$(a, b, c) = (2.45\ \mu m, 1.7\ \mu m, 1.7\ \mu m)$
Position of the particle's center	$(x_0, y_0, z_0) = (0\ \mu m, 0\ \mu m, 13.5\ \mu m)$
Position of the ellipsoid's center	$(x_0, y_0, z_0) = (0\ \mu m, 0\ \mu m, 3\ \mu m)$
w_0 in for ellipsoid	0.75 um
w_i in for ellipsoid	0.35 um
Radius of the ring	2.3 μm
Position of the ring's center	$(x, y, z) = (-1.6\ \mu m, 0\ \mu m, 0\ \mu m)$
Number of laser beams for a half ring	30
Ambient temperature	293.15 K
Thermal conductivity (Water)	COMSOL Built-in Library
Coefficient of thermal expansion (Water)	COMSOL Built-in Library
Heat capacity at constant pressure (Water)	COMSOL Built-in Library
Density (Water)	COMSOL Built-in Library
Dynamic viscosity (Water)	COMSOL Built-in Library
Thermal conductivity (PDMS)	$0.15\ (W \cdot (m \cdot K)^{-1})$
Heat capacity at constant pressure (PDMS)	$1460\ (J \cdot (kg \cdot K)^{-1})$
Density (PDMS)	$970\ (kg \cdot m^{-3})$
Thermal conductivity (Ellipsoid)	$0.6\ (W \cdot (m \cdot K)^{-1})$
Heat capacity at constant pressure (Ellipsoid)	$1308\ (J \cdot (kg \cdot K)^{-1})$
Density (Ellipsoid)	$1100\ (kg \cdot m^{-3})$
**FDTD**	
Software	Lumerical, Matlab
Total power	0.8384 mW
Dimension of simulation domain	$7\ \mu m * 7\ \mu m * 10\ \mu m$
Refractive index of solution	1.338
Refractive index of Ellipsoid	1.4
Boundary conditions	PML
Simulation time	1000 fs
Size of the ellipsoid	$(r_1, r_2, r_3) = (2.45\ um, 1.7\ um, 1.7\ um)$
Wavelength	660 nm

Other Supplementary Materials for this manuscript including the following:

Movie S1. Arbitrary-axis rotation and real-time switching of a PDMS particle.

Movie S2. Arbitrary-axis rotation and real-time switching of a yeast cell.

Movie S3. Arbitrary-axis rotation and real-time switching of an algae cell.

Movie S4. Reorientation of a yeast cell under half-ring illumination on a bare glass substrate.

References

1 Liu, Q. & Prosperetti, A. Wall effects on a rotating sphere. *Journal of Fluid Mechanics* **657**, 1-21, doi:10.1017/S002211201000128X (2010).
